# The Knowledge and Health Belief Model of Osteoporosis Prevention Among Females in Saudi Arabia

**DOI:** 10.7759/cureus.75350

**Published:** 2024-12-08

**Authors:** Yousria Badawy, Ali A Bin Yameen, Mohammed Alasri, Nawaf A Alamri, Khalid Alrifai

**Affiliations:** 1 Family Medicine, Ibn Sina National College for Medical Studies, Jeddah, SAU; 2 Faculty of Medicine, Ibn Sina National College for Medical Studies, Jeddah, SAU

**Keywords:** barriers, benefits, cues to action, females, health belief model, knowledge, osteoporosis, self-efficacy, severity, susceptibility

## Abstract

Background

Osteoporosis is the most common bone disease in humans, representing a major public health problem affecting women more commonly. The prevalence of osteoporosis in Saudi Arabia is high among females. Thus, the knowledge and Health Belief Model (HBM) of osteoporosis, which can be used to gain an understanding of health behaviors and reasons for non-compliance to osteoprotective recommendations, is necessary. This study aimed to investigate the knowledge and constructs of the HBM for osteoporosis prevention among females in Saudi Arabia.

Methodology

This cross-sectional study investigated the knowledge and HBM which includes perceived susceptibility and severity, perceived benefits, barriers, self-efficacy, and clues to action for preventing osteoporosis among females in Saudi Arabia. The study population consisted of females living in Saudi Arabia aged 18 years or more and not having osteoporosis. The convenient consecutive non-probability sampling technique was used to recruit 406 females aged 18 years or more using an online form. In addition to content validity and Cronbach’s alpha for overall reliability, Bloom’s cut-off scale was utilized to evaluate the knowledge level and HBM.

Results

The study revealed a moderate level of knowledge of all the constructs of HBM except that of perceived barriers which was low. All the constructs of the HBM were significantly and positively correlated with knowledge level except perceived barriers which was significantly and negatively correlated with knowledge level. Many participants received cues to act, such as not being able to afford treatment or not having anybody to take care of them while they were sick.

Conclusions

The Saudi female population’s general knowledge of osteoporosis and HBM was moderate, while that of perceived barriers was low.

## Introduction

Osteoporosis is a disease characterized by the deterioration of bone tissue, disruption of bone microarchitecture, and low bone mass, compromising bone strength and increasing the risk of fractures [1]. Osteoporosis, the most common bone disease in humans, represents a major public health problem that is more common in women and older people. However, it is a silent disease until fractures occur, causing significant health problems and even death [2].

Previous estimates showed that the disease affects around 200 million female patients globally with 8.9 million secondary bone fractures [3]. The prevalence of osteoporosis in Saudi Arabia is estimated at 34%-39.5% among the female population [4]. Although exposure to the sun is abundant in Saudi Arabia, vitamin D deficiency is a common characteristic among Saudi patients, especially females [5].

Several factors are associated with an increased risk of osteoporosis-related fractures. These include female sex, Asian or Caucasian race, advancing age, family history of osteoporosis, or fragility fractures. Additionally, a low body mass index, menopause before the age of 45 years, prolonged amenorrhea unconnected to menopause, nulliparity, and prolonged lactation are other risk factors for osteoporosis. Furthermore, a calcium- and vitamin D-low diet, or poor intestinal calcium absorption, lactose intolerance, special habits such as excessive caffeine or alcohol consumption, smoking, sedentary lifestyle, and certain conditions, including prolonged treatment with thyroid hormones, glucocorticoids, anticonvulsants, aluminum antacids, and anticoagulants, can increase the risk of osteoporosis development [6].

As reported by the National Osteoporosis Foundation (2010), osteoporosis is incurable, although it can be prevented partly by increasing physical activity at all ages, adequate dietary calcium and vitamin D intake, and fall prevention. Cessation of smoking and reduction of alcohol consumption may also play a role. Additionally, weight-bearing exercises are among the most important preventive measures [7]. Previous studies showed that people of all ages lack knowledge about osteoporosis or do not recognize themselves as being at risk for developing bone loss and osteoporosis [8].

Using health behavior theories may increase the effectiveness of assessing osteoporosis knowledge and developing education efforts to address the issues related to the lack of awareness. The Health Belief Model (HBM) can be used to understand health behaviors and reasons for non-compliance to osteoprotective behaviors [9]. There are six constructs of the HBM [10]. The first four constructs were developed as the original HBM tenets. The last two were added as research on the HBM evolved. The six constructs are as follows: (1) perceived susceptibility, which refers to a person’s subjective perception of the risk of acquiring an illness or disease; (2) perceived severity, which refers to a person’s feelings on the seriousness of contracting an illness or disease (or leaving the illness or disease untreated); (3) perceived benefits, which refers to a person’s perception of the effectiveness of various actions available to reduce the threat of illness or disease (or to cure illness or disease); (4) perceived barriers, which refers to a person’s feelings on the obstacles to performing a recommended health action; (5) cue to action, which is the stimulus needed to trigger the decision-making to accept a recommended health action; and (6) self-efficacy, which refers to the level of a person’s confidence in their ability to successfully perform a behavior.

Hassan et al. (2021) reported that the level of knowledge and awareness about osteoporosis among the Saudi population is low and recommended further approaches to increase awareness among the general population to intervene against potential complications and enhance the prognosis [11]. Therefore, expanding the knowledge and raising the awareness of the public toward osteoporosis for proper prevention and management is essential. Moreover, ensuring that the vulnerable population understands the risk factors and can make an informed decision regarding their health-related behaviors to prevent such diseases is essential. This study aimed to assess the knowledge and identify the most common osteoporosis prevention health beliefs among females in Saudi Arabia.

## Materials and methods

Study design and setting

This cross-sectional study was conducted to evaluate knowledge and the HBM including perceived susceptibility and severity, perceived benefits and barriers, and self-efficacy, as well as cues to action (motivation) for osteoporosis prevention among females in Saudi Arabia. The study population comprised females aged ≥18 years without osteoporosis and living in Saudi Arabia. The study was conducted between January and December 2023. The period of data collection was from February to May 2023. Exclusion criteria were those aged below 18 years.

Sampling size and technique

A convenient consecutive non-probability sampling technique was used. The sample size was determined based on a single population formula using Epi-info version 7. The calculated sample size was 384 based on an estimated prevalence of 50% with a margin of error of 5%.

Data collection

A questionnaire was prepared by the researchers (English and Arabic), who checked its validity and reliability. The online questionnaire using Google Forms was sent to the participants. The questionnaire used was developed based on the HBM [10], including three parts. The first part includes sociodemographic, family, and fracture history data. The second part includes 20 questions on knowledge of osteoporosis (scored from 0 to 20). The third part includes questions on the components of the HBM questionnaire: seven questions on perceived susceptibility scored from 0 to 7 (the participant’s opinion about the chances of getting osteoporosis); seven questions on perceived severity scored from 0 to 7 (about osteoporosis-related complications); 11 questions on perceived benefits scored from 0 to 11 (about the benefits of behaviors preventing osteoporosis, such as physical activity and calcium intake); 12 questions on perceived barriers scored from 0 to 12 (including barriers to physical activity and consumption of calcium-rich foods), nine questions on self-efficacy scored from 0 to 9 (including the ability to exercise and have a proper diet); five questions on cues to action encouraging the subjects toward behaviors preventing osteoporosis. All questions are based on the standard five-point Likert scale ranging from strongly disagree to strongly agree (scored from 0 to 5). The scores for questions about cues to action are calculated as cumulative frequency.

Bloom’s cut-off scale was used to assess the level of knowledge and HBM [12]. The 80%-100% scores of total correct responses defined a high level of knowledge and perception of different items in the HBM. A score of 60%-79% was defined as the moderate level, whereas the low level was defined as a score of <60% of the total correct responses.

Three specialists and professionals (outside the team) in the field of health education and health promotion were consulted to assess the validity of the instrument. Experts were consulted to enhance the questionnaire’s relevance and clarity. Their feedback on the questionnaire’s usability, clarity, and additional data to improve the instrument’s rigor was incorporated into the final version. The experts recommended revising certain double-barreled questions and eliminating duplicated questions to improve the questionnaire’s effectiveness.

A pilot study was used to develop the plan and modify the main study to increase its quality and efficiency. The survey’s clarity, question order, and completion time were ensured through the pilot study, which was completed before the actual data collection. No modification of any questions was used after the pilot study was employed. All participants in the pilot study were included in the main study. Additionally, the pilot study was used to estimate internal consistency to assess reliability.

The overall reliability of the instrument was 0.859 based on the Cronbach’s alpha. Cronbach’s alpha was 0.733 for knowledge, 0.750 for perceived susceptibility, 0.785 for perceived severity, 0.904 for perceived benefits, 0.870 for perceived barriers, and 0.920 for self-efficacy. As the alpha values calculated for each component studied were >0.7, the reliability of the instrument was considered acceptable.

Statistical analysis

Data were collected and grouped using Microsoft Excel. Statistical analyses were performed using SPSS statistics software (IBM Corp., Armonk, NY, USA) to analyze the data. Different statistical methods were used to study different associations between the variables. P-values <0.05 indicated statistical significance.

Ethical considerations

The Ibn Sina National College of Medical Sciences approved this study (approval number: 02-12112023). All information obtained was kept strictly confidential. Consent for participation in the study was obtained from all participants and included in the data collection form.

## Results

Table 1 depicts the characteristics of females who participated in the online survey on their knowledge and health beliefs about osteoporosis. The study included 406 Saudi female participants. Participants were categorized into various age groups, with the age group of 41-50 years having the highest percentage of participants (23.4%). A small percentage (9.9%) of females were aged >61 years. Regarding education, the majority (72.4%) of participants held a university degree or above. On the contrary, a small percentage (1.7%) of participants were illiterate or had only elementary education. Regarding employment, roughly 72% of the females were unemployed. As for smoking, a minority (15.1%) of participants reported being current or former smokers. A history of a simple fracture was only reported by 10.1% of the females. Additionally, the history of first-degree family members who had a fracture because of a simple effort or positive family history of osteoporosis was identified in 29.3% and 28.3% of participants, respectively. Concerning the body mass index, less than two-thirds (59.9%) of participants were overweight or obese.

**Table 1:** Baseline characteristics of study participants (n = 406).

Characteristics		Frequency (n = 406)	Percentage (%)
Age categories in years	18–30	105	25.9
	31–40	87	21.4
	41–50	95	23.4
	51–60	79	19.5
	61–80	40	9.9
Level of education	Illiterate	2	0.5
	Primary school	5	1.2
	Secondary school	105	25.9
	University or above	294	72.4
Employment status	Unemployed	293	72.2
	Employed	13	27.8
Smoking history	Non-smoker	345	85.0
	Ex-smoker	23	5.7
	Smoker	38	9.4
History of a simple fracture	No	365	89.9
	Yes	41	10.1
Family history of a simple fracture	No	287	70.7
	Yes	119	29.3
Positive family history of osteoporosis	No	291	71.7
	Yes	115	28.3
Body mass index categories	Underweight	2	0.5
	Normal	161	39.7
	Overweight	129	31.8
	Obese	114	28.1

Table 2 shows that 52.0% and 16.7% of the participants had a moderate and high level of knowledge of osteoporosis, respectively. A moderate degree of perception was also displayed by around two-thirds of the individuals (68.0% and 61.1%) for perceived susceptibility or severity. However, high levels of benefit perception were reported by 49.0% of participants, whereas only a minority (3.2%) had a low level of benefit perception. In contrast, the majority (78.1%) of participants showed a low level of perceived barriers. Furthermore, a sizable share (60.8%) of participants demonstrated a moderate degree of self-efficacy.

**Table 2:** Knowledge and Health Belief Model data about osteoporosis among participants.

Knowledge and Health Belief Model categories of osteoporosis		Frequency	Percentage (%)
Knowledge score categories	High level of knowledge	68	16.7
	Moderate level of knowledge	211	52.0
	Low level of knowledge	127	31.3
Level of perceived susceptibility	High level of perceived susceptibility	36	8.9
	Moderate level of perceived susceptibility	276	68.0
	Low level of perceived susceptivity	94	23.2
Level of perceived severity	High level of perceived severity	83	20.4
	Moderate level of perceived severity	248	61.1
	Low level of perceived severity	75	18.5
Level of perceived benefit	High level of perceived benefit	199	49.0
	Moderate level of perceived benefit	194	47.8
	Low level of perceived benefit	13	3.2
Level of perceived barrier	High level of perceived barrier	9	2.2
	Moderate level of perceived barrier	80	19.7
	Low level of perceived barrier	317	78.1
Level of self-efficacy	High level of self-efficacy	106	26.1
	Moderate level of self-efficacy	247	60.8
	Low level of self-efficacy	53	13.1

As shown in Table 3, which illustrates the cues to action (the stimulus needed to trigger decision-making to accept a recommended health action) toward osteoporosis among study participants, cues to action (47.8%) were related to their inability to pay for the medication in nearly half of the participants. Furthermore, 36.5% of respondents reported having no one to look after them while they were unwell. Only 10.1% of the participants reported losing a close friend to osteoporosis-related problems. The majority of participants (58.6%) reported the negative experience of witnessing osteoporosis deteriorating a family member’s health. Additionally, 45.6% of the participants regularly reported watching TV messages that discussed osteoporosis and ways to prevent it.

**Table 3:** Cues to action toward osteoporosis among study participants.

Cues to action	Yes	No
Cannot afford medication for osteoporosis	52.2%	47.8%
Nobody to take care of me during the illness	63.5%	36.5%
A close friend died of osteoporosis complications	89.9%	10.1%
Watching osteoporosis weaken the health of a family member	41.4%	58.6%
Hearing frequent TV messages about osteoporosis and how to avoid it	54.4%	45.6%

As the study examined the constructs of knowledge and five HBM items separately, all questions per construct were computed into a total score. Table 4 shows the range of potential scores for each construct, as well as the mean and standard deviation. For knowledge, the possible score ranged from 0 to 20 with the mean and standard deviation of 14.07 and 2.866, respectively, indicating a moderate level of knowledge. For both constructs of perceived susceptibility and seriousness, the possible score ranged from 7 to 35 with a mean and standard deviation of 22.85 (4.032) and 24.00 (4.590), respectively. These values indicate a moderate level of perceived susceptibility, as well as the severity of osteoporosis as moderate. For the constructs of perceived benefits of behavior change, the possible score ranged from 11 to 55 with a mean and standard deviation of 42.79 and 6.327, respectively, indicating a moderate view of the construct. On the other hand, the possible score for perceived barriers to taking action ranged from 12 to 60 with a mean and standard deviation of 30.37 and 7.578, respectively. Thus, this score showed that participants had few perceived barriers. The last construct of the HBM, self-efficiency, showed a possible score ranging from 9 to 45 with a mean and standard deviation of 31.81 and 6.094, respectively, indicating moderate motivation for health.

**Table 4:** Osteoporosis knowledge and health belief scale: total constructs (n = 406).

Constructs	Mean	SD	Possible score	Interpretation related to osteoporosis
Knowledge score	14.07	2.869	0–20	Moderate knowledge level
Perceived susceptibility	22.85	4.032	7–35	Moderate level of perceived susceptibility
Perceived severity	24.00	4.590	7–35	Moderate level of perceived severity
Perceived benefits	42.79	6.327	11–55	Moderate level of perceived benefits
Perceived barriers	30.37	7.578	12–60	Low level of perceived barriers
Self-efficacy	31.81	6.094	9–45	Moderate level of self-efficacy

There was a positive association between knowledge and perceived susceptibility (r = 0.145), perceived severity (r = 0.160), perceived benefits (r = 0.299), and self-efficacy (r = 0.219) regarding the relationship between knowledge score and health belief constructs toward osteoporosis. On the other hand, perceived obstacles were negatively connected to knowledge (r = -0.155), indicating that high knowledge levels were linked to low levels of barriers (Table 5).

**Table 5:** Correlation between knowledge score regarding osteoporosis and health belief constructs among participants.

Knowledge score	Health belief constructs				
	Perceived susceptibility	Perceived severity	Perceived benefits	Perceived	Self-efficacy
Pearson correlation	0.145	0.160	0.299	−0.155	0.219
P-value	0.003*	0.001*	0.001*	0.002*	0.001*

Using the test of significance (chi-square), identifying the relationship between the degree of knowledge about osteoporosis and various constructs of the HBM was possible (Table 6). Overall, the greater levels of various components of the HBM showed a considerably higher level of knowledge, except for the perception of barriers, among which those with the highest levels of knowledge showed a low perception of barriers.

**Table 6:** Association of knowledge scores with different components of the Health Belief Model regarding osteoporosis.

Health Belief Model categories of osteoporosis		Knowledge score categories				
		High level of knowledge (n = 68)	Moderate level of knowledge (n = 211)	Low level of knowledge (n = 127)	Chi	P-value
		Frequency (%)	Frequency (%)	Frequency (%)		
Level of perceived susceptibility	High level of perceived susceptibility	3 (4.4)	18 (8.5)	15 (11.8)	18.48	0.001*
	Moderate level of perceived susceptibility	37 (54.4)	146 (69.2)	93 (73.2)		
	Low level of perceived susceptibility	28 (41.2)	47 (22.3)	19 (15.0)		
Level of perceived severity	High level of perceived severity	4 (5.9)	46 (21.8)	33 (26.0)	12.07	0.017*
	Moderate level of perceived severity	47 (69.1)	128 (60.7)	73 (57.5)		
	Low level of perceived severity	17 (25.0)	37 (17.5)	21 (16.5)		
Level of perceived benefit	High level of perceived benefit	22 (32.4)	97 (46.0)	80 (63.0)	29.28	0.001*
	Moderate level of perceived benefit	39 (57.4)	111 (52.6)	44 (34.6)		
	Low level of perceived benefit	7 (10.3)	3 (1.4)	3 (2.4)		
Level of perceived barriers	High level of perceived barriers	2 (2.9)	3 (1.4)	4 (3.1)	17.26	0.002*
	Moderate level of perceived barriers	24 (35.3)	29 (13.7)	27 (21.3)		
	Low level of perceived barriers	42 (61.8)	179 (84.8)	96 (75.6)		
Level of self-efficacy	High level of self-efficacy	9 (13.2)	59 (28.0)	38 (29.9)	10.78	0.029*
	Moderate level of self-efficacy	48 (70.6)	120 (56.9)	79 (62.2)		
	Low level of self-efficacy	11 (16.2)	32 (15.2)	10 (7.9)		

## Discussion

The prevalence of osteoporosis, a major public health problem affecting millions of people worldwide, increases with age, and the Kingdom of Saudi Arabia is no exception. Osteoporosis is characterized by low bone mass and microarchitectural degeneration of bone tissue. The main objective of this study was to assess the knowledge and health beliefs related to the prevention of osteoporosis among a sample of females aged ≥18 years without osteoporosis and living in Saudi Arabia. Particularly, the study investigated their knowledge of the preventive effects of sun exposure, vitamin D exposure, calcium intake, physical activity, tobacco use, body shape, and family history. It also evaluated the perceptions of the severity and susceptibility to osteoporosis, the perceived benefits and barriers to physical activity and calcium intake, and the corresponding motivation to introduce measures preventing osteoporosis [13].

Knowledge regarding osteoporosis is crucial in disease prevention [4]. When evaluating the study population’s knowledge about osteoporosis, more than half (52.0%) of the participants had a moderate level of knowledge. These findings support research from Riyadh, Saudi Arabia, done in 2016, which found that 60.66% of participants had a moderate level of knowledge regarding osteoporosis [13]. The fact that both samples were from the same population explains this finding. However, investigations among Egyptian and Pakistani women showed low levels of knowledge about osteoporosis [14,15]. Due to the high prevalence of osteoporosis among Saudi women, this discrepancy may be linked to efforts made in Saudi Arabia to avoid osteoporosis [4].

Perceived susceptibility in the current study suggested a moderate degree of perception, which was positively correlated with the level of knowledge. These findings are represented in the modest degree of both knowledge and perception of susceptibility. These results differ from some studies reporting low perceived susceptibility to osteoporosis [14,16-18]. This discrepancy may be attributed to the young age of the majority of the population. Furthermore, the researchers of those studies mentioned that low perceived susceptibility might stem from the misconception that osteoporosis is an inevitable part of aging. Based on the results of the current study, a moderate level of severity perception was reported, which was positively correlated with the level of knowledge. These findings may be explained by a significant proportion of participants having a history of first-degree family members who had a fracture because of a simple effort or positive family history of osteoporosis (29.3 and 28.3%, respectively). Hence, living with a relative having osteoporosis may increase the level of participants’ awareness that osteoporosis is a serious disease. These results were supported by some studies showing that osteoporosis was perceived as a serious disease [8,14].

Regarding perceived benefits, participants showed a moderate level of perceived benefits, which was positively correlated with their level of knowledge. Thus, there was sufficient belief in the benefits of calcium intake and physical activity for osteoporosis prevention. This finding agreed with the studies by Endicott [16] and Kashfi et al. [17]. Ensuring sufficient calcium and vitamin D either from food or supplements may help individuals achieve better bone health [19]. Physical activity is an important determinant of bone health among youths. The positive effects of physical activity on various aspects of bone health were illustrated in several studies [20,21].

The level of barriers to calcium intake and physical activity were low. This agrees with the findings of Endicott (2013) in the United States and Elsabagh et al. (2015) in Egypt [14,16]. In other words, participants have no barriers to calcium intake and physical activity, which is beneficial for osteoporosis prevention. These barriers to calcium intake and physical exercises were negatively correlated with knowledge in the current study in contrast to previous studies that found that the knowledge of osteoporosis does not correlate with the amount of physical activity or total calcium intake [8,18].

Self-efficacy criteria, as described in a study conducted in the United States, include the belief that one can initiate an activity, maintain an activity, and persist in performing an activity in the face of barriers [16,21]. Based on our results, the mean self-efficacy constructs scores were moderate and positively associated with the knowledge level. These results were relatively similar to the findings of Tsai (2008) in New Zealand [18] and Endicott (2013) in the United States [16]. A sufficient level of health motivation is an important trigger for the implementation of relevant osteoporosis prevention programs.

Action cues, which are social elements that are part of the HBM, are perceived social influences that impact behavior’s performance or non-performance. The individuals’ behaviors to avoid osteoporosis were influenced by these external influences. This demonstrates how well external cues function as information sources, encouraging eating and walking habits, and providing the tools and direction required to measure bone density. Our findings are in line with those from recent studies that have identified several action signals (i.e., triggers that initiate the decision-making to embrace a suggested health remedy) that are advantageous for osteoporosis [22]. Many research participants received cues to act, such as not being able to afford treatment or not having anybody to take care of them while they were sick.

The study results highlight the need for primary healthcare programs related to osteoporosis in Saudi Arabia, specifically targeting women. Thus, health education programs for the public in the media are mandatory. Training of medical professionals is also important, emphasizing the importance of exercise, adequate consumption of a diet rich in calcium and vitamin D, and how to suspect the illness. Furthermore, more research is needed to confirm the hypothesis that a higher level of understanding about osteoporosis may lead to better bone health in the Saudi population. Raising awareness among women is significant because, in addition to encouraging positive habits to prevent osteoporosis, they can also act as agents of awareness in society at large.

Study limitations

The variables under investigation did not exhibit a cause-and-effect relationship according to this cross-sectional study. Because the measure used to evaluate participants’ knowledge and opinions regarding osteoporosis was self-reported and subjective, it is easy for respondents to exaggerate or underestimate their ratings. Consequently, it may be assessed through larger-scale research using both quantitative and qualitative methodologies.

## Conclusions

The Saudi population’s general knowledge of osteoporosis and HBM was deemed moderate, apart from the level of perceived barriers, which was low. This conclusion is consistent with other studies performed in other cities around the kingdom, indicating that no significant steps have been taken to address the issue. The effectiveness of lifestyle changes and preventative actions all depend on having a solid understanding and awareness of the condition.
